# Serological Evidence of Human Infection with *Coxiella burnetii* after Occupational Exposure to Aborting Cattle

**DOI:** 10.3390/vetsci8090196

**Published:** 2021-09-16

**Authors:** Ana Rabaza, Federico Giannitti, Martín Fraga, Melissa Macías-Rioseco, Luis G. Corbellini, Franklin Riet-Correa, Darío Hirigoyen, Katy M. E. Turner, Mark C. Eisler

**Affiliations:** 1 Instituto Nacional de Investigación Agropecuaria, Plataforma de Investigación en Salud Animal, Estación Experimental La Estanzuela, Colonia 70000, Uruguay; ar16974@bristol.ac.uk (A.R.); fgiannitti@inia.org.uy (F.G.); mfraga@inia.org.uy (M.F.); mmaciasrioseco@gmail.com (M.M.-R.); luis.corbellini@ufrgs.br (L.G.C.); franklinrietcorrea@gmail.com (F.R.-C.); dhirigoyen@inia.org.uy (D.H.); 2 Bristol Veterinary School, University of Bristol, Langford House, Langford, Bristol BS40 5DU, UK; katy.turner@bristol.ac.uk; 3 California Animal Health & Food Safety Laboratory System, University of California-Davis, Tulare, CA 95616, USA; 4 Programa de Pós Graduação em Ciência Animal nos Trópicos, Faculdade de Veterinária, Universidade Federal da Bahia, Ondina, Salvador 40170-290, Brazil

**Keywords:** coxiellosis, indirect fluorescent antibody test (IFAT), occupational hazard, Q fever, zoonosis

## Abstract

Cattle are broadly deemed a source of *Coxiella burnetii*; however, evidence reinforcing their role in human infection is scarce. Most published human Q fever outbreaks relate to exposure to small ruminants, notably goats. Anti-phase II *C. burnetii* IgG and IgM were measured by indirect fluorescent antibody tests in 27 farm and veterinary diagnostic laboratory workers to ascertain whether occupational exposure to cattle aborting due to *C. burnetii* was the probable source of exposure. Four serological profiles were identified on the basis of anti-phase II IgG and IgM titres. Profile 1, characterised by high IgM levels and concurrent, lower IgG titres (3/27; 11.1%); Profile 2, with both isotypes with IgG titres higher than IgM (2/27; 7.4%); Profile 3 with only IgG phase II (5/27; 18.5%); and Profile 4, in which neither IgM nor IgG were detected (17/27; 63.0%). Profiles 1 and 2 are suggestive of recent *C. burnetii* exposure, most likely 2.5–4.5 months before testing and, hence, during the window of exposure to the bovine abortions. Profile 3 suggested *C. burnetii* exposure that most likely predated the window of exposure to aborting cattle, while Profile 4 represented seronegative individuals and, hence, likely uninfected. This study formally linked human Q fever to exposure to *C. burnetii* infected cattle as a specific occupational hazard for farm and laboratory workers handling bovine aborted material.

## 1. Introduction

*Coxiella burnetii* causes the zoonosis Q fever, a disease which typically occurs after the inhalation of aerosolised contaminated material from the placenta or birth fluids of ruminants following either abortion or normal delivery [1,2]. Evidence for transmission of *Coxiella burnetii* by ingestion of contaminated raw dairy products is equivocal; infection was reported after consumption of raw cow’s milk [3] and contaminated goat cheese [4,5], but neither clinical Q fever nor antibodies were detected following deliberate human consumption of unpasteurised contaminated milk [6]. Although there is a high global prevalence of *C. burnetii* in cattle [7] and cattle are widely considered a risk for Q fever, there is little, if any, formal evidence for the contribution of bovines to human infection. Most published reports of human outbreaks in Europe relate to exposure to small ruminants, notably goats, as the main source of human infection [8,9,10,11]. Evidence for the association of an outbreak of Q fever affecting 1300 people in southeast Poland with cattle was limited to the demonstration of specific antibodies in bovines [12,13]. Only weak serological evidence supported *C. burnetii* as the cause of bovine abortions that were epidemiologically associated with Q fever outbreaks in Germany and Poland, but other common bovine abortifacient agents such as *Neospora caninum* and *Brucella abortus* were not investigated [14,15]. We are not aware of any published data firmly linking Q fever to *C. burnetii*-positive bovine abortions.

Q fever is asymptomatic in approximately 60% of cases [16]. Acute clinical presentation includes a wide range of non-specific symptoms, while endocarditis and chronic fatigue syndrome are the principal chronic manifestations [2,17], particularly in people with pre-existing conditions (cardiopathies, aneurysms, immunocompromise or pregnancy) [18,19,20].

Variation in the lipopolysaccharides of the bacterial outer membrane results in antigenic phases that determine diverse types of anti-*C. burnetii* immunoglobulins (anti-phase I and anti-phase II IgM, IgG and IgA). During infection, phase II antigens appear to dominate immunoglobulin responses [21] and immunoglobulin profiling is used to characterise chronicity of exposure [22,23,24]. Immune responses to phase II antigens are characteristic of acute exposure, whereas anti-phase I titres characterise chronicity [25,26].

While no previous human Q fever outbreak has been firmly related to bovine abortion caused by *C. burnetii*, this study presents more substantial laboratory evidence for a specific occupational hazard for workers exposed to aborted cattle or handling material from bovine abortions.

## 2. Materials and Methods

### 2.1. Bovine Abortions and Window of Workers Exposure

Following an outbreak of bovine abortion in a dairy herd in Colonia Department, Uruguay, placentas and full-term foetuses from four aborting cattle were collected by farmworkers and submitted to the local veterinary diagnostic laboratory between 10 April and 2 June 2017. Bovine coxiellosis was confirmed on the basis of typical placental lesions on histopathology, with identification of intralesional *C. burnetii* antigen in trophoblasts by immunohistochemistry and PCR amplification of DNA, and other abortifacients of cattle were ruled out by comprehensive testing [27]. The outbreak of coxiellosis was notified to the local health authorities, which triggered an investigation by public health officials. Serological sampling of humans was conducted on 14 and 21 August 2017, i.e., 18.1 and 19.1 weeks following exposure. Serological testing was performed on 27 farm and laboratory workers directly or indirectly exposed to the aborting cattle, foetuses and placentas. None of these workers had been vaccinated against *C. burnetii*.

### 2.2. Farm and Laboratory Workers’ Data and Consent

Written consent was obtained from all patients and their information was anonymised. Records comprised demographic data such as age, gender, clinical findings obtained during a medical examination, pre-existing medical conditions and the individual laboratory indirect fluorescent antibody test (IFAT) results for anti-*C. burnetii* phase II IgM and IgG antibodies. Data were made available by explicit agreement of the workers who were assured of confidentiality. Details about medical treatments could not be accessed. No animal or human samples were collected or analysed expressly for this study and results of laboratory testing were evaluated for this study as a secondary analysis. The study was granted ethical approval by the ethical committee from the University of Bristol (Ref.95382/Id.342095).

### 2.3. Review of Case Records from the Veterinary Diagnostic Laboratory

Records of diagnoses made by the local veterinary diagnostic laboratory between 10 April 2016 and 21 August 2017 were examined to rule out other potential exposures of laboratory workers to *C. burnetii*.

### 2.4. Indirect Fluorescent Antibody Test

Serum samples were analysed for anti-*C. burnetii* phase II IgM and IgG antibodies by the Mayo Clinic Laboratory (Rochester, MN, USA) using the indirect fluorescent antibody test (IFAT) for anti-*C. burnetii* phase II IgM and IgG antibodies [28,29].

### 2.5. Statistical Analysis

Titres less than 1/16 in the IFAT for anti-*C. burnetii* phase II IgM and IgG antibodies were considered to be seronegative and those greater or equal to 1/16 were considered to be seropositive. The percentage of seropositivity was calculated as the number of seropositive individuals (titre ≥ 1/16) divided by the total number of workers tested. Phase II IgG to IgM ratios were calculated by dividing the IgG titre by the IgM titre. Univariable and multivariable analyses were conducted in which the IFAT status (seropositive or seronegative) was considered as the binary response variable. Gender (male and female), age group (21–30, 31–40 and >40) and work activity (farm and laboratory) were included as explanatory variables in univariable and multivariable logistic regression models used to gain insight into factors (and their interactions) influencing *C. burnetii* seropositivity and to calculate odds ratios (OR) and their confidence intervals (CI_95%_). Statistical analysis was performed using RStudio software [30].

## 3. Results

The study population comprised 27 individuals who worked either on the farm, in the laboratory or both. Twenty-three individuals conducted at least some of their work on the farm, these comprising thirteen farm workers, two veterinary practitioners and eight laboratory workers. Twelve individuals conducted at least some of their work in the veterinary laboratory, these comprising the eight laboratory workers, who also conducted some farm work, and four further laboratory workers who did not.

Ten of the 27 individuals had detectable titres of IgG antibody to *C. burnetii* phase II greater or equal to 1/16, and, of these, five also had detectable titres of IgM. Of the 23 conducting work on the farm, eight (34.8%) were IgG positive and four (17.4%) of these were also IgM positive. Seven of the twelve (58.3%) conducting lab work had detectable IgG titres, and four (33.3%) of these were also IgM positive, noting that eight individuals undertook both types of work (Table 1). The univariable odds ratios for conducting laboratory work were 5.6 (CI_95%_ 1.09–35.6, *p* = 0.039) for IgG seropositivity and 7.0 (CI_95%_ 0.853–150, *p* = 0.071) for IgM seropositivity, i.e., statistically significant for IgG and close to significance for IgM. The corresponding univariable odds ratios for conducting farm work were 0.533 (CI_95%_ 0.055–5.13, *p* = 0.566) for IgG seropositivity and 0.632 (CI_95%_ 0.060–14.6, *p* = 0.726) for IgM seropositivity, i.e., not significant in either case.

The rate of seropositivity was twice as high in female workers (5/9, 55.6%; univariable odds ratio 3.25, CI_95%_ 0.623–18.7; *p* = 0.162) as in males (5/18, 27.8%) for IgG, but only slightly higher in females (2/9, 22.2%; univariable odds ratio 1.43, CI_95%_ 0.161–10.7; *p* = 0.729) than males (3/18, 16.7%) for IgM, in neither case statistically significant differences.

Rates of IgG seropositivity in age groups 21–30 (4/8, 50%) and 31–40 (5/10, 50%) were identical and these were collapsed into a single category. Seropositivity in individuals less than or equal to 40 years old (9/18, 50.0%; univariable odds ratio 8.00, CI_95%_ 1.12–165; *p* = 0.037) was significantly higher than those greater than 40 (1/9, 11.1%) for IgG whereas for IgM, seropositivity in individuals less than or equal to 40 (4/18, 22.2%; univariable odds ratio 2.29, CI_95%_ 0.275–48.9; *p* = 0.468) was not significantly higher than those greater than 40 (1/9, 11.1%). Four of the five (80%) individuals seropositive for IgM were in the 31–40 year age group, which was significant (univariable odds ratio for age 31–40 compared to all other ages 10.7, CI_95%_ 1.27–233, *p* = 0.0283).

Seropositivity levels for IgG were similar in symptomatic and asymptomatic (4/11, 36.4%) individuals for both IgG (symptomatic 6/16, 37.5%; asymptomatic 4/11, 36.4%; univariable odds ratio for symptoms 1.05, CI_95%_ 0.213–5.4; *p* = 0.952) and IgM (symptomatic 3/16, 18.8%; asymptomatic 2/11, 18.2%; univariable odds ratio for symptoms 1.04, CI_95%_ 0.143–9.12; *p* = 0.970).

In the multivariable analysis for IgG seropositivity addition of none of the terms farm work, age group, gender or symptoms improved upon the univariable model with laboratory work as the sole explanatory variable (likelihood ratio test *p* > 0.4 for all), suggesting this was already the minimum adequate model (*p* = 0.039). However, lab work was apparently confounded with age, with 11 of the 12 individuals conducting lab work being under 40 years of age. On collapsing the age group to just two levels, as already noted, nine of the eighteen (50%) workers less or equal to 40 years of age were seropositive for IgG, but just one of the nine (11.1%) workers over 40 years of age was IgG seropositive (odds ratio and CI_95%_ as above), and this was also the only individual in the over 40 age group conducting lab work; contrastingly, there were broadly similar numbers of IgG seropositives (6/9, 66.6%) and seronegatives (5/9, 55.6%) conducting lab work in the 40 and under age group (Fisher’s exact test *p* ≈ 1).

There were too few IgM seropositive individuals (n = 5) for a meaningful multivariable analysis; it was, however, noteworthy that four of the five conducted farm work, four conducted lab work, with three conducting both farm and lab work, and that all four of those IgM positive individuals conducting field work were in the 31–40 age category, the remaining IgM seropositive individual, who conducted only lab work, being in the 41–50 age category.

**Figure 1:**
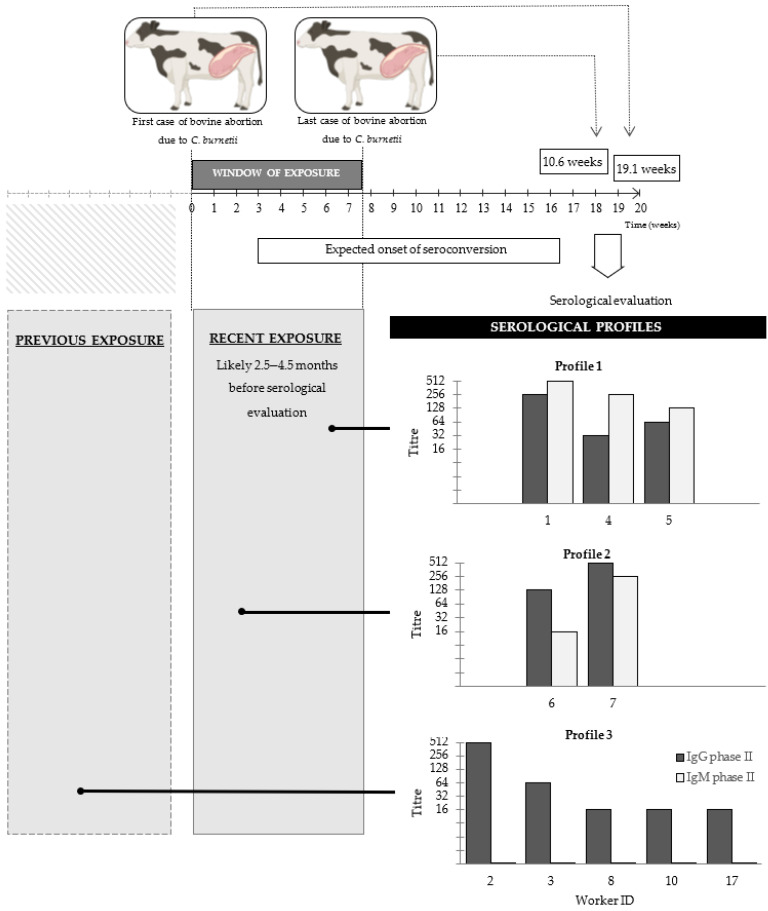
Timeline of the Q fever outbreak in cattle and serological investigations in farm and laboratory workers. Time zero was the date of the first case of bovine abortion. The window of exposure of farm and laboratory workers (when abortions occurred, and aborted materials were collected and submitted to the local veterinary diagnostic laboratory for diagnostic work-up) lasted for 7.7 weeks (10 April to 2 June 2017). The time course of the seroresponse was estimated based on published observations (Todkill et al., 2018). Serological sampling of humans was conducted on 14 and 21 August 2017, i.e., 18.1 and 19.1 weeks following the opening of the exposure window. Serological profiles are based on anti-*C. burnetii* phase II IgG and IgM levels measured by indirect fluorescent antibody test (IFAT). The profile of immunoglobulins was used to ascertain how recently they were likely to have been infected: Profile 1: both isotypes detected, IgM titre > IgG titre—very recent; Profile 2: both isotypes detected, IgM titre < IgG titre; Profile 3: IgG detected but not IgM. Profile 4: neither IgM nor IgG detected (data not shown). Reciprocal titres are shown.

When anti-*C. burnetii* phase II IgM and IgG titres were interpreted in conjunction, four distinct serological profiles could be identified among the workers (Figure 1). Five workers (IDs. 1, 4, 5, 6 and 7) had detectible titres (at least 1/16) of both IgM and IgG. Three of these five workers (IDs. 1, 4 and 5) whose IgM titres were higher than their IgG titres were classified as Profile 1, while the two workers (IDs. 6 and 7) whose IgG titres were higher than their IgM titres were classified as Profile 2. Five workers (IDs 2, 3, 8, 10 and 17) showed only IgG phase II titres with no detectable levels of IgM and were classified as Profile 3. Finally, 17 workers (IDs 9, 11–16, 18–27), in whom neither IgM nor IgG titres were detected, were classified at Profile 4.

Six of the ten seropositive workers manifested a variety of non-specific symptoms, whereas the remaining four seropositive workers remained asymptomatic. Among those with clinical disease, sweating, fever, fatigue and odynophagia were the most frequently reported. Most of the symptomatic workers (IDs 1, 3, 5 and 6) manifested clinically by middle–late May, i.e., three months before their serologic evaluation. Two workers (IDs 2 and 10) reported non-specific symptoms occurring around mid–late July (a month before serological examination). None of the seropositive workers had any medical condition known to predispose them to subsequent medical complications [18,19,20]. A review of the seventeen seronegative workers’ medical records revealed that ten presented some non-specific flu-like symptoms, whereas the other seven of these seronegative workers remained asymptomatic. The proportion of symptomatic individuals was very similar among seropositive (6/10, 60.0%) and seronegative workers (10/17, 58.8%) (univariable analysis, *p* ≈ 1.00).

The local veterinary laboratory examined submissions from fifty bovine and five ovine cases of abortion. Each case comprised either the foetus, the placenta or both. All cases were routinely examined for gross and histologic lesions, and cultured onto MacConkey and blood agar, Skirrow’s medium and *Leptospira* medium-based EMJH agar. Additionally, *Neospora caninum*, *Campylobacter* spp., *Tritrichomonas foetus*, Bovine parainfluenza virus 3 and Bovine viral diarrhoea virus 1 were investigated by immunohistochemistry, direct immunofluorescence, dark-field microscopy examination or PCR. None of these 55 foetuses presented any typical lesions leading to suspicion of coxiellosis. The cause of the bovine abortion was diagnosed in 25 of these cases (25/50). Most were diagnosed as infectious abortions (23/25) including agents such as *N. caninum* (11/23), *Campylobacter fetus* subsp. *venerealis* (1/23) and Bovine parainfluenza virus 3 (1/23), as well as opportunistic agents (8/23). In two out of the five cases of ovine abortion, *Campylobacter jejuni* and *Campylobacter fetus* were detected by PCR, while the other three cases remained undiagnosed.

## 4. Discussion

The chronology of serological responses and the immunoglobulin classes involved were investigated in a group of workers exposed to bovine abortions caused by *C. burnetii* to ascertain whether these aborted cattle were likely to have been the source of human exposure. Surprisingly, given the importance assigned to Q fever as a zoonotic disease globally, there is an extreme paucity of evidence in accessible peer-reviewed literature associating Q fever with cattle. Most of the publications that have investigated human Q fever outbreaks conducted serological assessments in people, followed by, at most, a description of the epidemiological link between people and cattle (such as visits to the affected herd, regular consumption of raw milk or unpasteurised dairy products, or occupational exposure); a few complemented this with PCR evaluations. Some studies also conducted serological evaluation in animals. However, serological approaches are not particularly informative in cattle as seroconversion can occur without bacterial shedding, and animals can remain seropositive long after overcoming the infection; conversely cattle can shed *C. burnetii* before the development of detectable antibodies and may even shed the agent without ever seroconverting [31]. In contrast, our study provides more convincing evidence that exposure to aborted dairy cattle and their abortion materials is an occupational hazard for the acquisition of Q fever.

In our study, based on the dates of the first and last known bovine abortion cases, the window of exposure to *C. burnetii* was estimated to comprise a timeframe of 54 days (Figure 1). On this basis, at the time of serological investigation, the workers had been exposed for no longer than 134 days (19.1 weeks) after contact with the first bovine case and no less than 74 days (10.6 weeks) after contact with the last bovine case. Thus, the workers were exposed to *C. burnetii* 74–134 days prior to serological examination. The incubation period of Q fever (exposure to disease onset) is pathogen dose-dependent, estimated at between 7 and 32 days (one to five weeks) [32], and seroconversion takes place roughly 14–28 days (two to four weeks) later [21,32,33]. Hence, seroconversion may be expected after 21 days and, almost certainly, no later than 60 days (three to nine weeks) after exposure. On this basis, we estimate that the seropositive workers in our study may have seroconverted between a theoretical minimum of 74 − 60= 14 days and a maximum of 134 − 21= 113 days prior to serological examination. Hence, serological investigation was conducted at least 1.6 weeks, and possibly as much as 16.1 weeks, after seroconversion would be expected based on their exposure to bovine cases.

The profile of immunoglobulins reactive against *C. burnetii* antigens was used to provide insight into the timing of the acquisition of infection based on the known kinetics of antibody development in clinical Q fever [34,35]. In three cases (IDs 1, 4 and 5), IgM titres were higher than IgG titres suggesting exposure had been recent and coinciding with the latter stages of the known window of exposure to aborting cattle. Two other workers (IDs 6 and 7) were also seropositive for both antibody isotypes but had higher IgG titres than IgM; this may have reflected slightly less recent exposure, perhaps earlier on during the known window of exposure and possibly associated with the first bovine case. The minimum and maximum times between exposure to aborting cattle and the serological evaluation of workers (74–134 days) was entirely consistent with this abortion outbreak being the source of the human infections. The IgG anti-phase II concentration tends to exceed that of IgM anti-phase II on average by about 4.5 days after the onset of the serological response, which equates to 25.5 days after exposure to *C. burnetii* [35]. Given that seroconversion may be expected 21–60 days after exposure, in our study we would expect seroconversion to have preceded serological testing by a minimum of 14 days. While this is a little longer than the estimated average time of 4.5 days from seroconversion to the point at which the IgG titre exceeds that of IgM, for some workers to have IgM titres higher than IgG at the time of testing was entirely consistent with the aborting cattle indeed being the source of exposure.

The IgG phase II antibody has a greater half-life than IgM phase II, with persistence up to 2.5 years, making it an indicator of past infection [35]. In our study, five seropositive workers (IDs 2, 3, 8, 10 and 17) had IgG phase II antibody titres but no detectable IgM phase II, suggesting that exposure might have been long before the known recent outbreak of bovine abortion, and those workers may have had a previous exposure that preceded the documented bovine outbreak.

In addition to the profile of immunoglobulins, the IgG/IgM ratio can be used as a rough estimator of the time after infection and can be used to discern between infection within three months and infection more than six months ago [36]. The IgG/IgM ratio is about 0.1 early after the onset of symptomatology, approximates to 1.0 within the first 100 days and is greater than 10 during the following 100 days. In our study, the IgG/IgM ratio ranged between 0.1 and 0.5 in worker IDs 1, 4 and 5, and between 2 and 8 in worker IDs 6 and 7. This evidence supports recent exposure and is entirely consistent with known exposure to aborting cattle 74–134 days prior to serological analysis.

Two of the workers (IDs 2 and 10) had serological profiles suggestive of long past infection; they reported non-specific symptoms that were likely due to another aetiology as they occurred long after probable exposure to *C. burnetii*. Likewise, symptoms reported by seronegative workers could be due to other seasonal illnesses, and their responses on symptomatology could have been affected by their awareness of the investigation (Hawthorne bias).

The odds of *C. burnetii* seropositivity in laboratory workers, including those also undertaking occasional field activities, were greater than those for field workers for both anti-phase II IgG (OR 5.6 CI_95%_ 1.09–35.6) and anti-phase II IgM (OR 7.0 CI_95%_ 0.853–150). Most of the farmworkers did not assist at calving and, hence, were exposed to *C. burnetii* infection indirectly, e.g., through urine and faeces. Considering that shedding of *C. burnetii* by cows through these routes is scarce and intermittent [37], field workers would have faced a repeated but low-level bacterial challenge. In contrast, people engaged in laboratory activities but without direct contact with farm animals might have been exposed to a high bacterial burden through the handling of abortion material infrequently or even on just a single occasion. Despite the suggestion of a protective role of female hormones such as *β*-estradiol [38], infection rates were similar in male and female workers. Nor was an age-related increase in Q fever seropositivity observed in our study, as has been reported elsewhere [39]. For IgG, there were a far greater number of seropositives in the 40 and under age group (9/18) than in older individuals (1/9). Unfortunately, conducting lab work was confounded with age and it was difficult to be certain whether conducting lab work or being of an age 40 and under was the most important determinant of IgG seropositivity. The observation elsewhere that seropositivity tends to increase with age [39] would indeed support lab work as being the more important of the two in this instance.

Other than the previously documented cases of bovine abortion due to coxiellosis [27], none of the bovine or non-bovine abortions routinely analysed by the local veterinary laboratory revealed macroscopic or histologic evidence suggestive of *C. burnetii* infection. Although other sources of *C. burnetii* exposure in laboratory workers beyond the analysed bovine outbreak cannot be altogether excluded, the known exposure to well-documented cases of bovine abortion caused by coxiellosis appears to be a far more likely and plausible source of infection for the human cases described in this study.

This study had a number of limitations that could be considered in future work aiming at furnishing further evidence for *C. burnetii* infection in humans exposed to infected bovines or their abortion products. While the aetiology of the bovine abortions themselves was confirmed using molecular methods (PCR) as well as histopathology and immunohistochemistry [27], the subsequent human infections documented here were confirmed only by serology; confirmation by molecular methods [40] would have strengthened this evidence. The bovine outbreak is the most probable source of infection for laboratory workers and veterinarians, but other sources cannot be fully excluded. Furthermore, the extent to which the symptomatology described by the patients was related to Q fever is unclear. While the symptoms described and their chronology were consistent with acute infection with *C. burnetii* [41], we were unable to demonstrate a statistical association between symptoms and serological responses in the Phase II IFA for either IgG or IgM (*p* > 0.95). This might have been possible with a larger number of cases, but this was a study of a naturally occurring disease event and the sample size was not within our control. In this study, we used a titre of 1/16 or greater in the Phase II IFA as the seropositivity threshold for both IgG and IgM, as this was considered above the reference level by the testing laboratory (Mayo Clinic Laboratories), and, indeed, some authorities have used even lower IFA titres in epidemiological studies [42]. We, nevertheless, performed a sensitivity analysis and re-analysed the data using a more conservative seropositivity threshold of 1/32, with little change in the overall implications of the results. Using this higher cut-off value, although there were fewer Phase II IgG positives overall (seven rather than ten), the association with lab work was even stronger, having an even higher odds ratio (14.0, CI_95%_ 1.85–297) and a lower p-value (*p* < 0.01); for IgM there were four rather than five positives overall and the revised odds ratio (4.67, CI_95%_ 0.507–103) remained suggestive but non-significant (*p* = 0.179). Lastly, the persistence of phase II IgM must be considered in the interpretation of results when investigating acute Q fever, particularly in endemic, post-epidemic and late epidemic contexts [29]. This is not likely to be a significant limitation to the present study as, although there has been no centralised system of recording and few data are available, to the best of the authors’ knowledge, Q fever cases have been reported only sporadically since the first local outbreak was documented in 1956 [43].

## 5. Conclusions

In conclusion this epidemiological investigation, the first closely linking Q fever to bovine abortion, provides novel serological evidence of *C. burnetii* exposure in people working in direct contact with either aborted cattle or their foetuses, placentas and vaginal discharges. Cattle aborting due to *C. burnetii* should not be underestimated as a potential hazard and possible source of human infection. Q fever should be considered in the spectrum of diseases in patients with an epidemiological link with animals, or with occupational-related exposure, especially those with fever of unknown origin. Vaccination should be considered for people at risk of Q fever through occupational exposure.

## Figures and Tables

**Table 1:** Anti-*Coxiella burnetii* phase II IgM and IgG titres, IgG to IgM ratio, demographic factors, background data of workers and potential exposure based on work activity.

Worker ID	Age Range (Years)	Gender	Type of Work	Exposure Category	IgG Phase II Titre *	IgM Phase II Titre *	Phase II IgG/IgM Ratio	Symptomatic ^†^
1	41–50	M	Bacteriologist	Laboratory	1/256	1/512	0.5	Yes
2	21–30	F	Veterinary diagnostician	Farm and laboratory	1/512	<1/16	-	Yes
3	21–30	F	Veterinary diagnostician	Farm and laboratory	1/64	<1/16	-	Yes
4	31–40	F	Veterinary diagnostician	Farm and laboratory	1/32	1/256	0.1	No
5	31–40	F	Farm veterinarian	Farm	1/64	1/128	0.5	Yes
6	31–40	M	Veterinary diagnostician	Farm and laboratory	1/128	1/16	8	Yes
7	31–40	M	Laboratory technician	Farm and laboratory	1/512	1/256	2	No
8	31–40	F	Veterinary diagnostician	Laboratory	1/16	<1/16	-	No
9	41–50	M	Farmworker	Farm	<1/16	<1/16	-	No
10	21–30	M	Farmworker	Farm	1/16	<1/16	-	Yes
11	31–40	F	Laboratory technician	Laboratory	<1/16	<1/16	-	Yes
12	61–70	M	Farmworker	Farm	<1/16	<1/16	-	No
13	21–30	M	Farmworker	Farm	<1/16	<1/16	-	Yes
14	21–30	M	Farm veterinarian	Farm	<1/16	<1/16	-	Yes
15	31–40	M	Farmworker	Farm	<1/16	<1/16	-	Yes
16	51–60	M	Farmworker	Farm	<1/16	<1/16	-	Yes
17	21–30	M	Farmworker	Farm	1/16	<1/16	-	No
18	41–50	M	Farmworker	Farm	<1/16	<1/16	-	Yes
19	31–40	F	Farmworker	Farm	<1/16	<1/16	-	No
20	51–60	M	Farmworker	Farm	<1/16	<1/16	-	No
21	41–50	M	Farmworker	Farm	<1/16	<1/16	-	No
22	31–40	F	Veterinary diagnostician	Farm and laboratory	<1/16	<1/16	-	Yes
23	31–40	M	Veterinary diagnostician	Farm and laboratory	<1/16	<1/16	-	No
24	21–30	M	Veterinary diagnostician	Farm and laboratory	<1/16	<1/16	-	Yes
25	51–60	M	Farmworker	Farm	<1/16	<1/16	-	No
26	41–50	M	Farmworker	Farm	<1/16	<1/16	-	Yes
27	21–30	F	Laboratory technician	Laboratory	<1/16	<1/16	-	Yes

* For both antibody isotypes, titres of <1/16 were considered negative; M: male; F: female. ^†^ At least one suggestive symptom reported.

## Data Availability

Data available upon request due to restrictions e.g., privacy or ethical.
